# Practitioner Review: Attention‐deficit hyperactivity disorder and autism spectrum disorder – the importance of depression

**DOI:** 10.1111/jcpp.13678

**Published:** 2022-08-16

**Authors:** Anita Thapar, Lucy A. Livingston, Olga Eyre, Lucy Riglin

**Affiliations:** ^1^ Division of Psychological Medicine and Clinical Neurosciences, MRC Centre for Neuropsychiatric Genetics and Genomics Cardiff University School of Medicine Cardiff UK; ^2^ Wolfson Centre for Young People's Mental Health Cardiff University Cardiff UK; ^3^ Neuroscience and Mental Health Research Institute Cardiff University Cardiff UK; ^4^ Institute of Psychiatry, Psychology and Neuroscience King's College London London UK

**Keywords:** ADHD, autism spectrum disorders, depression, risk factors, assessment

## Abstract

Young people with neurodevelopmental disorders, such as attention‐deficit hyperactivity disorder (ADHD) and autism spectrum disorder (ASD), show high rates of mental health problems, of which depression is one of the most common. Given that depression in ASD and ADHD is linked with a range of poor outcomes, knowledge of how clinicians should assess, identify and treat depression in the context of these neurodevelopmental disorders is much needed. Here, we give an overview of the latest research on depression in young people with ADHD and ASD, including possible mechanisms underlying the link between ADHD/ASD and depression, as well as the presentation, assessment and treatment of depression in these neurodevelopmental disorders. We discuss the implications for clinicians and make recommendations for critical future research in this area.

## Introduction

Attention‐deficit hyperactivity disorder (ADHD) and autism spectrum disorder (ASD) are neurodevelopmental disorders (American Psychiatric Association (APA), 2013; Thapar, Cooper, & Rutter, 2017) that represent common reasons for referral to specialist services. Whilst neurodevelopmental disorders (NDDs) in themselves are impairing, individuals are at substantially elevated risk of developing mental health problems, of which depression is one of the most common (Erskine et al., 2016; Ghaziuddin, Ghaziuddin, & Greden, 2002). Given that impairment and outcomes are much worse for young people with NDDs who develop depression (e.g. Kraper, Kenworthy, Popal, Martin, & Wallace, 2017; Rice et al., 2019), timely recognition, prevention and effective treatment of depression is important. Strikingly, depression in NDDs is rarely addressed in clinical guidance and research priority documents. Here, we consider recent research on depression in ADHD and ASD (see Supporting Information for search strategy) and discuss the implications for practitioners. Although ADHD and ASD show strong clinical and aetiological overlap, they have distinct features (Thapar et al., 2017), and most research has focused on one condition alone. Thus, we will consider each in turn, but draw out commonalities and distinctions where possible.

## How common is depression in ADHD and ASD?

Depression rates vary widely across studies, likely due to differences in sample characteristics (e.g. age and measurement approaches). In ADHD, rates of depression range from 0% to 44.5% and are more than 5 times higher compared to individuals without ADHD (Angold, Costello, & Erkanli, 1999). The cumulative incidence of depression in individuals with ADHD followed up to age 19 has been estimated at 23% (Yoshimasu et al., 2012), and 44% of individuals with ADHD have experienced a depressive episode before age 30 (Meinzer, Pettit, & Viswesvaran, 2014). In ASD, one recent meta‐analysis estimated 10.6% prevalence of depression, which is four times that seen in typically developing youth, and the prevalence only increases further as individuals transition into adulthood (Hudson, Hall, & Harkness, 2019).

Depression is more common in females, but it is unclear whether the same female bias is observed in ADHD and ASD, given that these disorders show a male excess (Thapar et al., 2017). In ADHD, some studies suggest depression is more common in females (e.g. Jensen & Steinhausen, 2015), however others find no gender difference (e.g. Yoshimasu et al., 2012). In ASD, evidence is also mixed, but the most recent meta‐analysis suggests depression rates are comparable between males and females (Hudson et al., 2019).

## Why are young people with ADHD/ASD at risk for depression?

**Figure 1:**
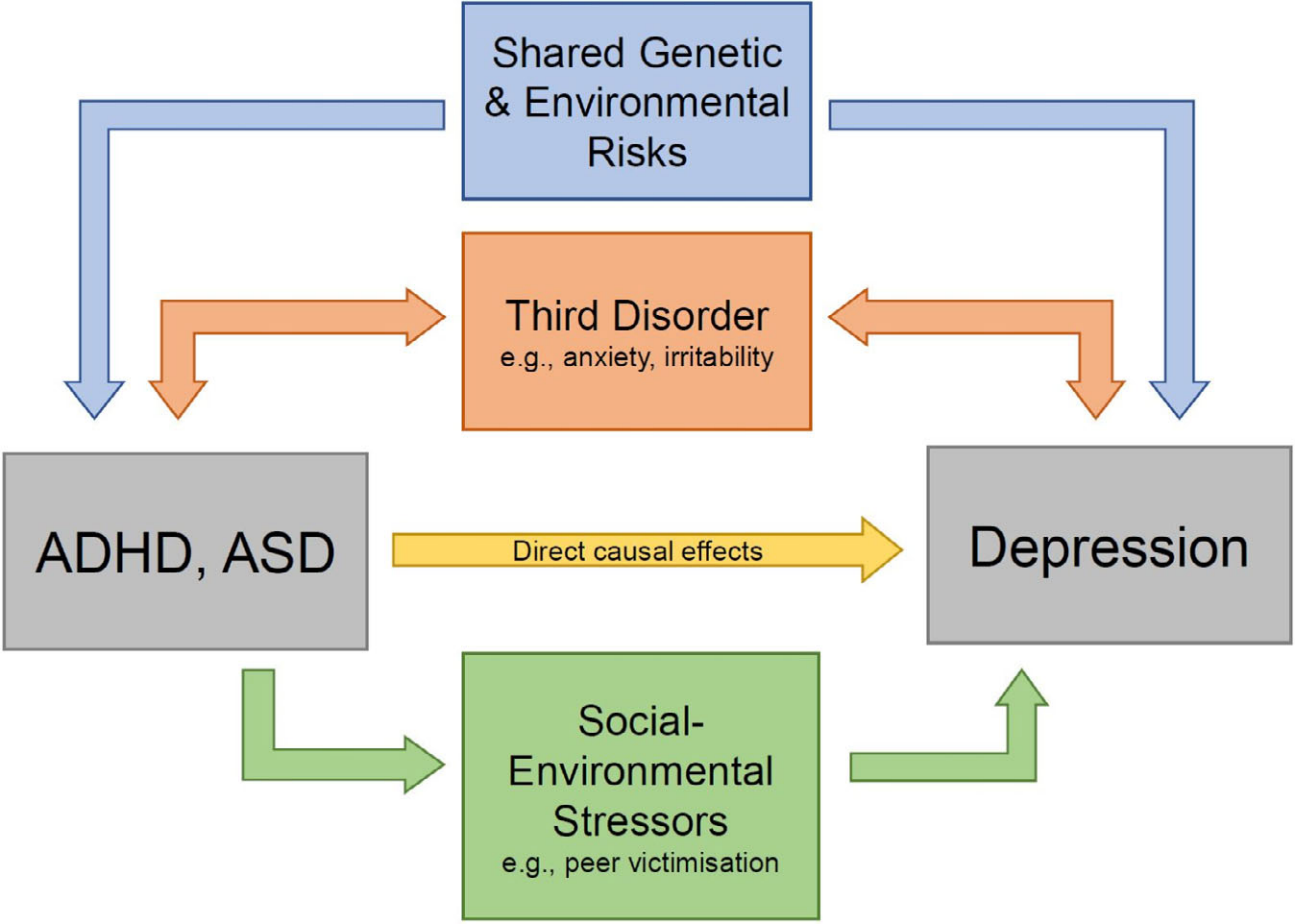
Possible mechanisms linking ADHD/ASD and depression. ADHD and ASD may be linked with depression due to: (1) An artefact of shared symptoms or medication side effects (see Figure 2); (2) shared genetic and environmental risks between ADHD/ASD and depression; (3) direct causal effects of ADHD/ASD on depression; (4) indirect effects via social‐environmental stressors; and/or (5) ADHD/ASD and depression both being associated with a third disorder or difficulty [Color figure can be viewed at wileyonlinelibrary.com]

**Figure 2:**
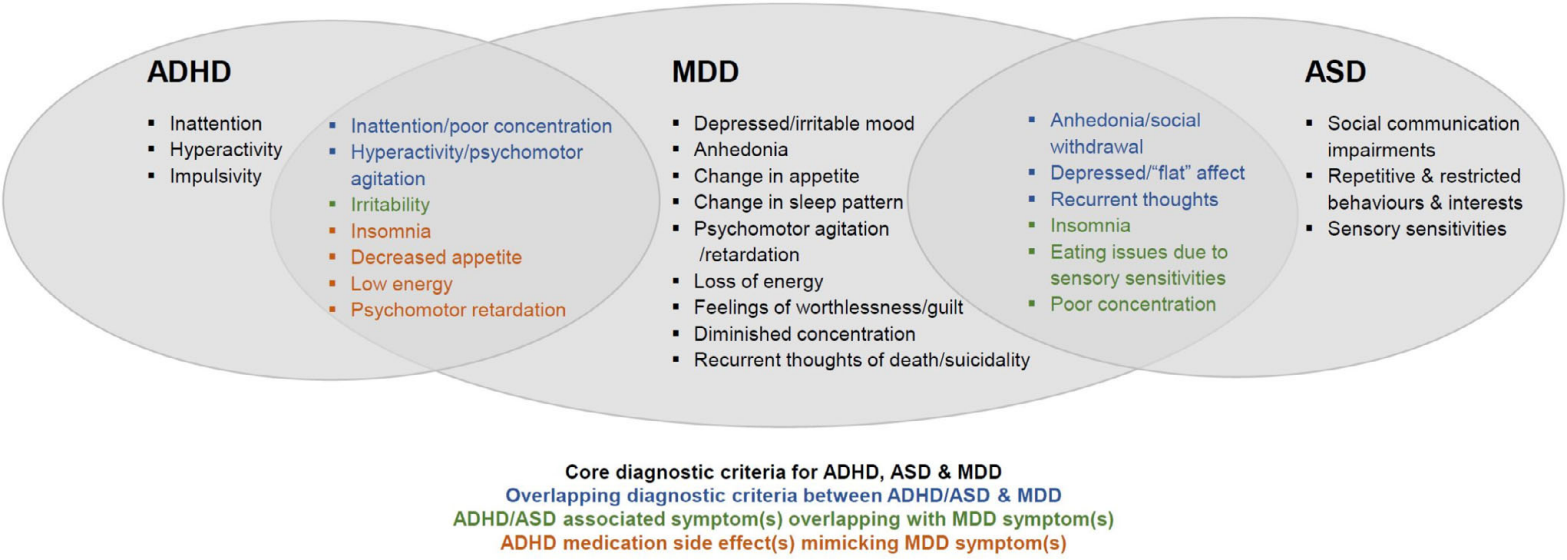
Diagnostic criteria for ADHD, ASD and major depressive disorder (MDD). ADHD and ASD may look similar clinically to MDD due to: (1) Overlapping core diagnostic criteria, for example, poor concentration is a symptom of both ADHD and MDD; (2) ADHD/ASD associated symptoms overlapping with MDD symptoms, for example, insomnia (a symptom of MDD) is highly prevalent in ASD; and/or (3) ADHD medication side effects mimicking MDD symptoms, for example, decreased appetite [Color figure can be viewed at wileyonlinelibrary.com]

There are multiple possible explanations for the links between NDDs and later depression (Figure 1; Caron & Rutter, 1991). First, associations could be an artefact of shared symptoms or medication side effects (Figure 2). However, studies have shown that associations remain even after these factors are accounted for (e.g. Biederman, Faraone, Mick, & Lelon, 1995). Second, links could be explained by shared aetiology, whereby the same genetic and/or environmental risks contribute to both ASD/ADHD and depression. Third, the clinical features of ADHD/ASD could serve as *direct* risks for depression, or fourth, lead *indirectly* to depression via social‐environmental stressors. Finally, links could be explained by a third disorder that commonly accompanies NDDs.

### Shared genetic and environmental risks

ADHD and ASD are highly heritable (Thapar, 2018; Thapar & Rutter, 2021), and genetic factors modestly contribute to depression. Thus, depression could be more common in NDDs if the same genetic liability contributes to both disorders. Indeed, family studies have shown that parents and other biological relatives of those with ADHD or ASD exhibit heightened rates of depression (Chen et al., 2019; Wang et al., 2022). Twin studies further suggest that familial links between ADHD/ASD and later depression are explained mainly by shared genetic liability (e.g. Stern et al., 2020). Genome‐wide association studies that identify common genetic variants at a molecular level also show a shared genetic aetiology; of all psychiatric disorders, depression shows the highest genetic correlation with both ADHD (Demontis et al., 2019) and ASD (Grove et al., 2017). However, there is little evidence that the same environmental risks contribute to NDDs and depression, as those that contribute to depression, such as bullying and stressful life events (Thapar, Collishaw, Pine, & Thapar, 2012), tend to occur after the typical age‐at‐onset for NDDs.

Of course, substantive genetic contributions to ASD/ADHD do not rule out social‐environmental stressors promoting later depression. Young people with ADHD or ASD are exposed to more social adversities than their peers. This may occur because environments are not independent of one's characteristics or genetic liability. Gene–environment correlation arises when a person's genetic liability to a disorder is also associated with exposure to environmental risks, thereby resulting in a ‘double whammy’ risk effect (Rutter, 2015). For example, one study of children adopted at birth found that child ADHD genetic liability, indexed by biological parent ADHD, led to increased adoptive mother–child hostility (Harold et al., 2013). Maltreatment is also more common in children with NDDs (Clayton, Lee, Cheung, Theule, & Henrikson, 2018; Hoover & Kaufman, 2018). One hypothesis is early childhood maltreatment or trauma cause ADHD/ASD, as well as later depression. However, studies that take into account genetic liability suggest that NDDs increase the likelihood of childhood maltreatment (Stern et al., 2018), rather than maltreatment causing NDDs (Dinkler et al., 2017), although one study could not rule out a causal effect (Capusan et al., 2016).

More recently, other studies have used a composite measure of common genetic risk variants (polygenic risk scores; PRS) as an indicator of genetic liability for NDDs and observed that ADHD and ASD PRS are associated with child maltreatment (e.g. Ratanatharathorn et al., 2021) and broader childhood adverse experiences (e.g. Zwicker et al., 2020). On balance, it seems probable that genetic liability for NDDs and their phenotypic manifestations increase the risk for maltreatment and some of the social‐environmental stressors known to increase depression risk.

### Direct causal effects

A second possible mechanism is that ADHD or ASD directly causes depression. Despite strong evidence that ADHD and ASD are associated with later depression, observational designs alone cannot disentangle different explanations for these associations. Treatment‐based designs provide one approach to examine whether ADHD or ASD have direct causal effects on depression. If they do, then effective treatment of ADHD/ASD that reduces core symptoms should reduce risk of depression. However, a recent systematic review found no meta‐analyses of randomised control trials (RCTs) in which depression was an outcome of interventions for ADHD/ASD (Correll et al., 2021). An alternative, quasi‐experimental design, is to investigate within‐individual effects of ADHD/ASD treatment on depression. One such study found a 20% lower rate of depression when individuals were, versus were not, receiving ADHD medication (Chang, D'Onofrio, Quinn, Lichtenstein, & Larsson, 2016). This is consistent with a causal effect of ADHD on depression. However, these designs have limitations, and are less easily applied in the absence of disorder‐specific medication, such as for ASD.

Mendelian randomisation (MR) is an alternative method, utilising genetic data to infer causality (Hemani, Bowden, & Davey Smith, 2018), although it requires many assumptions. One recent MR study found evidence consistent with a causal effect of ADHD genetic liability on major depressive disorder (MDD; Riglin et al., 2020), although findings were less consistent for broader depression. To our knowledge, there are no MR studies investigating the association between ASD and depression. Overall, across treatment‐based and MR designs, two studies suggest that ADHD may have a causal effect on depression, but no conclusions can be made about ASD.

### Social‐environmental mechanisms

Another explanation is that NDDs are linked with later depression indirectly via social‐environmental stressors.

In ADHD, studies examining family relationships have found the association between childhood ADHD and depression is partially mediated by parenting (e.g. Ostrander & Herman, 2006) and, for inattention symptoms, by parent–child relationship difficulties (Humphreys et al., 2013). One longitudinal study found that for boys, ADHD symptoms were associated with a steeper increase in negative mother–child relationships across adolescence compared to girls, and that this partly mediated the association between ADHD symptoms and subsequent depression (Meinzer, Felton, Oddo, Rubin, & Chronis‐Tuscano, 2020).

Peer relationships also are implicated. Population‐based studies have observed that friendship quality and peer problems (Powell, Riglin, et al., 2021), and being victimised by peers (Roy, Hartman, Veenstra, & Oldehinkel, 2015), partially mediate the association between ADHD and depression symptoms. One study, however, did not find strong evidence of mediation by declining best‐friend relationship quality across adolescence (Meinzer et al., 2020). Interestingly, Powell, Riglin, et al. (2021) found evidence that the association between ADHD and depression symptoms – mediated via friendship quality – attenuated in children with more positive parent–child relationships. This highlights that positive social relationships in one context may be able to compensate for poor social relationships in others.

Competency‐based (Cole, Martin, & Powers, 1997) and dual‐failure (Patterson & Stoolmiller, 1991) models of depression suggest that ADHD‐linked impairments may affect sense of competency/failure, which thereby increases risk of depression. For example, one cross‐sectional study of Japanese children found that ADHD (inattention) symptoms were associated with depression via low self‐esteem (Kita & Inoue, 2017). In addition, several studies have investigated academic competence as a pathway from ADHD to depression. While some found academic ability, indexed by test/exam scores, to partially mediate the association between ADHD symptoms in childhood and subsequent depression (Herman, Lambert, Ialongo, & Ostrander, 2007; Powell et al., 2020), others have not found strong evidence for this link (Humphreys et al., 2013).

In ASD, research also highlights negative social‐environmental experiences, such as bullying and traumatic events, as a route to later depression. In a population‐based study, Rai et al. (2018) found that being bullied during adolescence partially mediated the relationship between autistic (social communication) difficulties at age 7 and depression at age 18. Further, bullied autistic children showed elevated depressive symptoms across adolescence, compared to non‐bullied autistic and typically developing children. Cross‐sectional studies have also implicated peer victimisation (e.g. Chou, Wang, Hsiao, Hu, & Yen, 2020), which, similar to ADHD, may in part be buffered by parental support (Wright, 2017). A few studies have found less stressful family environments are associated with fewer depressive symptoms in autistic youth (e.g. Greenlee, Winter, & Marcovici, 2020), although there is less research focus on family environments in ASD compared to ADHD.

One prominent explanation for how ASD promotes depression is that depression arises from dysfunctional interpersonal relationships (Smith & White, 2020). Supporting this, evidence suggests that more severe autistic symptoms (Hedley, Uljarević, Foley, Richdale, & Trollor, 2018) and poor quality of social interaction (Dallman, Bailliard, & Harrop, 2022) are associated with greater depression. Further, reduced success with social relationships in ASD may lead to depression via loneliness. Indeed, Hedley et al. (2018) found that among autistic adolescents and adults, loneliness partly mediated the relationship between a lack of social support networks and depression.

Overall, research indicates that there are numerous social‐environmental factors that may mediate risk of depression in young people with ADHD and ASD. However, most evidence from the aforementioned studies is cross‐sectional, thus it is unclear if any of these factors play a causal role.

### A third disorder or difficulty

An alternative mechanism is that NDDs are associated with depression due to a third disorder or difficulty that commonly accompanies ASD/ADHD.

Childhood anxiety disorders are important predictors of future depression and common in ADHD, and thus may explain the association between ADHD and depression (Meinzer et al., 2014). One meta‐analysis of general population studies found that anxiety explained the association between ADHD and depression (Angold et al., 2009). Other studies have found that the association between ADHD and depression attenuates when adjusting for anxiety, oppositional defiant, and conduct disorders (e.g. Copeland, Shanahan, Erkanli, Costello, & Angold, 2013; Ford, Goodman, & Meltzer, 2003), however some studies still find a robust association after accounting for comorbid psychiatric disorders (e.g. Meinzer et al., 2013). Substance misuse is another comorbidity associated with ADHD and depression (e.g. Groenman, Janssen, & Oosterlaan, 2017), although whether it is a route to depression in ADHD is currently unclear. In terms of ASD, anxiety disorders are highly prevalent (Copeland et al., 2013), but whether anxiety explains the link between ASD and depression has not to our knowledge been directly tested.

Beyond comorbid psychiatric disorders, there are transdiagnostic difficulties that might promote the link between NDDs and depression. Emotional dysregulation is a common difficulty in NDDs and associated with depression (Cai, Richdale, Uljarević, Dissanayake, & Samson, 2018; Shaw, Stringaris, Nigg, & Leibenluft, 2014). Research suggests it mediates the link between ADHD and depression cross‐sectionally and longitudinally (Seymour et al., 2012; Seymour, Chronis‐Tuscano, Iwamoto, Kurdziel, & MacPherson, 2014). A closely related problem, irritability – difficulty regulating anger – is also strongly associated with ADHD and may be a route to later depression. In one population cohort, irritability explained almost half of the association between a broad range of childhood neurodevelopmental difficulties (including ASD and ADHD) and depression (Eyre et al., 2019). Additionally, sluggish cognitive tempo (SCT) – daydreaming, inattention, and lethargy – which is associated with but distinct from ADHD (Barkley, 2013), is also linked with depression (Smith, Zald, & Lahey, 2020). Whether SCT plays a direct role in promoting depression in ADHD requires investigation.

Finally, alexithymia – difficulty identifying one's own emotions – is highly prevalent in ASD (Kinnaird, Stewart, & Tchanturia, 2019) and also associated with depression (Bloch, Burghof, Lehnhardt, Vogeley, & Falter‐Wagner, 2021). Thus, heightened alexithymia could contribute to depression in ASD. For example, Morie, Jackson, Zhai, Potenza, and Dritschel (2019) found a serial relationship between alexithymia and emotional regulation partly mediated the association between ASD and depression in adults. Alexithymia may also be relevant to ADHD, but has been less studied as a pathway to depression.

## Presentation of depression in ADHD and ASD

### Timing and severity

When depression is comorbid with ADHD, it is more severe, has an earlier age‐at‐onset and worse prognosis than in the typically developing population. Purper‐Ouakil et al. (2017) found that adults with MDD who retrospectively reported childhood ADHD symptoms had greater depression symptoms and poorer global functioning, compared to those without childhood ADHD. Further, a recent study of women with recurrent depression found that those with broadly defined ADHD, versus not, had an earlier age of depression onset and a higher rate of receiving non‐first‐line antidepressants (Powell, Agha, et al., 2021). This suggests that in some individuals, underlying ADHD may be masked by depression. Prospective population‐based studies also suggest that childhood ADHD is associated with a younger depression age‐at‐onset (Jaffee et al., 2002; Rice et al., 2019).

In ASD, depression onsets in adolescence at a similar age to the typically developing population (Gotham, Brunwasser, & Lord, 2015; Gundel, Pedersen, Munk‐Olsen, & Dalsgaard, 2018; but see Rice et al., 2019), and rates increase across adolescence, particularly for autistic girls. Gotham et al. (2015) found that autistic boys had greater depression symptoms compared to autistic girls at age 13, but autistic girls' symptoms showed an upward trajectory, so that the sex difference was absent by age 21. There is also emerging evidence that ‘late diagnosed’ autistic young people may be at risk for more severe depression. In one cohort study, autistic girls diagnosed with ASD ‘late’ (aged 14–17) showed the greatest risk of later developing depression compared to autistic boys and girls diagnosed in childhood (Gundel et al., 2018). Hosozawa, Sacker, and Cable (2021) found a similar link between late diagnosis in adolescence and later depression, but no sex difference. Interestingly, previous depression diagnoses are highly prevalent among individuals diagnosed with ASD in adulthood (Fusar‐Poli, Brondino, Politi, & Aguglia, 2022), suggesting that like ADHD, ASD might be misdiagnosed as depression or depression might mask underlying ASD. Further research is needed to understand how common it is to miss NDDs in the context of depression, perhaps especially in females.

### Symptom presentation

Depression in ADHD and ASD may present differently to depression in the general population.

In ADHD, research suggests a similar factor structure and relative frequency of depression symptoms among those with and without ADHD (Fraser et al., 2018; Joseph et al., 2019). However, Diler et al. (2007) found that, among young people with ADHD, some depression symptoms (e.g. anhedonia, suicidal thoughts, psychomotor retardation) discriminated those with MDD from those without MDD, whereas others did not (e.g. irritability, reduced sleep, appetite, and concentration). Therefore, certain depressive symptoms may be more indicative of a depressive episode in young people with ADHD, although further research is needed. Additionally, young people with ADHD are impulsive and show elevated rates of self‐harm and suicidal behaviour, which may sometimes be the primary presentation of depression in ADHD. Further, irritability – a common feature of both ADHD and depression – may be present. However, ADHD‐related irritability typically onsets early, whereas irritability in depression will be accompanied by other depression symptoms and typically arise later in development (Riglin et al., 2019).

In ASD, some studies have found a similar factor structure for depression symptoms in people with and without ASD (e.g. Uljarević et al., 2018). However, one recent network analysis found that depression in ASD was particularly characterised by insomnia and restlessness (Montazeri, de Bildt, Dekker, & Anderson, 2020), suggesting these might be prominent features of depression in ASD. It is also proposed that there are ‘atypical’ presentations of depression that are more specific to ASD, for example, reduced engagement with special interests and self‐care (Pezzimenti, Han, Vasa, & Gotham, 2019). However, beyond clinical observations, there is a lack of systematic investigation into ASD‐specific manifestations of depression.

## Assessment of depression in ADHD and ASD

Although early detection is pivotal, there are numerous barriers to effectively assessing depression in NDDs. First, symptoms of ADHD and ASD overlap with depression symptoms, making it difficult to distinguish between the disorders (Figure 2). For example, difficulty concentrating and restlessness – core symptoms of ADHD – are also symptoms of depression. And irritability – a symptom of adolescent depression – is an associated feature of ADHD. ADHD medication also commonly results in side effects that mimic depression. For example, methylphenidate, the first‐line treatment for ADHD in young people (The National Institute for Health and Care Excellence (NICE), 2018) can cause decreased appetite and insomnia, which are diagnostic criteria for MDD (APA, 2013).

In ASD, individuals can exhibit social withdrawal, which overlaps with anhedonia in depression. Insomnia, another depression symptom, is also highly frequent in ASD (Mazzone, Postorino, Siracusano, Riccioni, & Curatolo, 2018), and autistic people can experience eating difficulties due to sensory sensitivities (Baraskewich, von Ranson, McCrimmon, & McMorris, 2021), which may be mistaken for depression‐related decreased appetite. One way to help differentiate between ADHD/ASD and overlapping depression symptoms is to consider age‐at‐onset of symptoms and evidence of a change in symptoms. NDDs have an earlier age‐at‐onset with symptoms persisting over time, whereas depression onset is later and symptoms are more episodic.

A second challenge is that many commonly used depression measures (e.g. Moods and Feelings Questionnaire) have not been validated for young people with NDDs and therefore may not be as accurate. For example, overlap between ADHD/ASD and depression may result in elevated depression scores. For ADHD, no specific tools have been developed, although it has been suggested that the Child Behaviour Checklist and the Strength and Difficulties Questionnaire (SDQ) may be useful for screening for depression (Biederman, Monuteaux, Kendrick, Klein, & Faraone, 2005; Coutinho, Farias, Felden, & Cordeiro, 2021). In ASD, the SDQ has been shown to be an effective screening tool for emotional difficulties in autistic children (Findon et al., 2016), however the SDQ is not specific to depression, capturing anxiety too. A French tool for screening depression in ASD has recently been developed (Bellalou, Downes, & Cappe, 2021), although to our knowledge is yet to be translated into English. Overall, much more rigorous evaluation of the suitability of common depression measures for use in ASD and ADHD is needed.

The third challenge is that depression may be hard to assess due to differences between various informants. Young people with NDDs may under report their own depressive symptoms. For example, there is evidence of higher parent‐ versus self‐rated depression scores in both ADHD (e.g. Fraser et al., 2018) and ASD (e.g. Schwartzman & Corbett, 2020). In ASD, alexithymia and social communication can make it difficult for individuals to reflect on or explain their internal states verbally, especially those with co‐occurring intellectual disability. Arguably, parents may be a more useful informant. However, it is also possible that they may over report symptoms, or even miss certain symptoms in the presence of other behavioural difficulties. Parents will likely have less insight into their children's internal states as they move through adolescence and into early adulthood, as reflected in reduced concordance between self and parent ratings over development (e.g. in ASD, Andersen, Hovik, Skogli, & Øie, 2017). Taken together, rater differences suggest the need for multiple informants when assessing for depression in NDDs. For those with ASD and intellectual disability, parent and teacher reports of behaviour change and clinician observation are particularly important.

Overall, the assessment of depression in ADHD and ASD can be complicated by numerous factors. Depression screening tools may not be as accurate, so careful clinical assessment is necessary, obtaining information from both the young person and parent, together with observations from school, in relation to new changes in behaviour and functioning.

## Effectiveness of standard depression treatments

### Psychological therapies

Psychological therapies are effective and recommended for treating depression in young people (NICE, 2019), but there is less evidence regarding their effectiveness for treating depression in those with NDDs.

In ADHD, numerous psychological therapies for depression have been evaluated. One meta‐analysis examined studies targeting emotional symptoms (depression, anxiety and emotional dysregulation) in children and adults with ADHD, using cognitive behavioural therapy (CBT), mindfulness‐based therapy (MBT), parent training, social skills training and (dialectical) behavioural therapy (Guo, Assumpcao, & Hu, 2022). Results suggested that, in children, parent training was effective in reducing depressive symptoms, social skills training was effective for emotional dysregulation, but CBT did not have a significant effect. In adults, CBT was effective for reducing depression, emotional dysregulation and anxiety. Additionally, Lopez et al. (2018) found that when CBT was used to treat core ADHD symptoms in adults, depression symptoms improved when compared to treatment as usual. Therefore, while there is evidence for use of CBT for treating depression in adults with ADHD, this does not seem to be generalisable to young people.

Psychological therapies for depression in ASD also have been evaluated. A meta‐analysis of CBT in young people and adults with ASD found a small‐to‐medium effect size when treating affective disorders (Weston, Hodgekins, & Langdon, 2016). However, results varied according to who rated the outcome, with significant effects seen for informant‐ and clinician‐reported outcomes, but not for self‐reported outcomes. Additionally, most of the studies in the meta‐analysis targeted anxiety rather than depression, and of those studies examining depression, only one included young people (McGillivray & Evert, 2014). In that study, a group CBT intervention was compared to waiting list controls in young people aged 15–25 with ASD, finding a significant reduction of depression symptoms in the intervention compared to waiting list group for those who initially scored highly on depression.

In terms of MBT, evidence of it as an effective depression treatment in ASD is limited to adults (e.g. Sizoo & Kuiper, 2017). Other studies examining the effect of psychological therapies have targeted core symptoms of ASD or anxiety, but included depression as a secondary outcome (e.g. Bemmer et al., 2021; Schiltz et al., 2018). These findings suggest that treating possible mediators of the association between ASD and depression (e.g. social functioning, social anxiety) can improve depression symptoms, but these effects may not generalise to MDD.

The choice of psychological therapy for depression in young people with ASD may also depend on the individual's cognitive and verbal ability. For those who are minimally verbal or have intellectual disability, approaches focusing on behavioural components of CBT, such as behavioural activation, may be a better choice (White et al., 2018). However, full‐scale RCTs to evaluate the effectiveness of such interventions are yet to be published. Psychological therapies also have been adapted to be more accessible and effective for those with ASD (White et al., 2018), for example, focusing on developing emotional awareness, slower pace, inclusion of special interest, reduced use of metaphors, structured sessions, use of visuals and additional time for practice.

Overall, there is some evidence to suggest psychological therapies can be effective in treating depression in young people with ADHD or ASD, but these may work better in older adolescents and young adults. Modified CBT approaches may be effective in autistic young people, with evidence for family‐based approaches in young people with ADHD. Nevertheless, further research on the effectiveness of psychological therapies directly targeting depression in both ADHD and ASD is needed.

### Medication

To the best of our knowledge, there are no published RCTs on medication for treating depression in NDDs. There is also no evidence to guide clinicians on the most effective medications for ADHD in the presence of comorbid depression.

For ADHD, there are consensus guidelines on the assessment and treatment of comorbid depression in adult life, some of which are relevant to young people (McIntosh et al., 2009). These guidelines recommend prioritising the treatment of the most severe or impairing condition first. If ADHD is the most concerning disorder and depression is only mild, effective treatment of ADHD is the priority. As ADHD medications, including stimulants, can disrupt sleep and mood, those with depression will need careful monitoring. If depression is severe and impairing, then the priority is to treat depression first using national guidelines before treating ADHD symptoms with medication. Fluoxetine is the only antidepressant currently licenced for use in children and adolescents in the UK, although observational and genetic studies suggest that depression with ADHD may be more likely to be treatment resistant (e.g. Fabbri et al., 2021). Beyond fluoxetine, there is modest evidence for the efficacy of escitalopram, sertraline, and duloxetine in youth depression (Hetrick et al., 2021). Venlafaxine and paroxetine are not recommended for young people. Unfortunately, there is no evidence to guide clinicians on antidepressants specifically in the context of ADHD.

The risk of attempted suicide is higher in those with ADHD (Ljung, Chen, Lichtenstein, & Larsson, 2014), so careful monitoring and supervision are needed in those with depression, whether or not medication is used. Clinicians should also be mindful that, after treatment with selective serotonin reuptake inhibitors, some young people with ADHD with depression could potentially switch to hypomania, given the known association between ADHD and early‐onset bipolar disorder, especially in the offspring of those with bipolar disorder (e.g. Kim et al., 2015). However, this is not a contra‐indication to initiate antidepressant medication. Although it is unclear how common antidepressant‐induced hypomania is in NDCs, and this will represent a small sub‐group, clinicians should carefully monitor young people if there is a family history of bipolar disorder.

For ASD, while psychosocial interventions are considered most important, medication may be required if depression is severe. There is no clear guidance on what works best in ASD based on RCTs (NICE, 2012). Given this, as for ADHD, currently it is recommended that clinicians follow national guidelines in relation to treating depression. However, one set of guidance suggests starting on a very low dose, titrating slowly, setting treatment targets, checking whether there is a family history of bipolar disorder, and being mindful of side effects, including aggression, impulsivity and disinhibition (Pezzimenti et al., 2019). Also, as in ADHD, there is a heightened risk for suicide in ASD (Costa, Loor, & Steffgen, 2020), so this also needs to be carefully monitored.

## Clinical implications

Despite growing research on the links between NDDs and depression, there is relatively little to guide clinicians in practice. For those who see young people with ADHD and/or ASD, accompanying irritability, alexithymia, anxiety, a family history of depression and exposure to social‐environmental stressors (e.g. bullying or maltreatment), are indicators of a heightened risk for depression. Social support in one context may help buffer stressors in another. While there are no prevention studies of depression in young people with ADHD or ASD, research to date suggests that effective treatment of core ADHD symptoms, treatment of parent depression (while being aware of the possibility of an underlying NDD in the parent too), reducing exposure to social stressors and increasing social support (at least in one context) may help disrupt the link with later depression. Overall, it is important to address parent mental health and the social context, comorbid difficulties as well as the core deficits of ADHD and ASD. Clinicians also need to be aware that early‐onset or difficult to treat depression, especially in girls and young women, could be masking an underlying NDD that has been missed.

There is limited evidence on how best to assess or treat depression effectively in ADHD and ASD. Therefore, we suggest that clinicians follow existing guidelines for identifying and treating depression in young people (i.e. NICE, 2019), but carefully consider adaptations as needed. For example, clinicians may need to interpret questionnaire measures with some caution and rely on direct clinical assessments as well as information from multiple informants. Similarly, usual depression treatment approaches may need to be adapted depending on the young person, their difficulties and strengths, and their social context. For example, psychological therapies may need to emphasise behavioural aspects, especially in younger individuals, and antidepressant treatment titrated and monitored more cautiously than in the typically developing population.

## Future research

This review has highlighted many research gaps. One striking gap is that research has focused exclusively on ADHD or ASD despite their established overlap. Given that, until DSM‐5 was published, research into ASD will have excluded those with a diagnosis of ADHD, many studies may have excluded young people who typify clinic populations where comorbidities are common. A second gap is that although young people with ADHD/ASD are at elevated risk for depression, it remains unknown how best to screen for and assess depression in the context of NDDs. Standard measures of depression need to be validated, particularly for young people, and if necessary, adapted for those with NDDs. A third major gap is there is little evidence to guide treatment choices. Thus, more RCTs of interventions for prevention and treatment of depression in ADHD and ASD are needed. Additionally, whether the aetiology, presentation or treatment of depression in NDDs differs at different developmental time points (e.g. childhood versus adolescence) requires investigation. Finally, most people with a NDD do not develop depression, and if they do, outcomes can be variable. More work is needed to identify how to increase resilience in those with NDDs and whether it is possible to identify those at highest risk of poorer outcomes, including developing a different mental disorder such as bipolar disorder or attempting suicide.

## Supporting information


**Table S1.** Search criteria for literature search.
