# Supplementary material for: Practitioner Review: Attention‐deficit hyperactivity disorder and autism spectrum disorder – the importance of depression
Source: J Child Psychol Psychiatry. 2022 Aug 16;64(1):4–15. doi: 10.1111/jcpp.13678 (PMC10087979; doi:10.1111/jcpp.13678)
Supplement: Supplementary file 1 — Table S1. Search criteria for literature search. [file JCPP-64-4-s001.docx]

**Supporting Information – Thapar *et al*.**

Table S1 presents the search criteria for our literature search, conducted on PubMed between July and September 2021. All search terms were specified for Title/Abstract, in the past 5 years and in English only.

Supplementary references informed the review, but were included in the main text due to word count restrictions.

**Table S1**

*Search Criteria for Literature Search*

| Topic | Search Terms | Number of Results |
| --- | --- | --- |
| Prevalence | (“ADHD” OR “attention deficit hyperactivity disorder” OR “hyperkinetic”) AND (“depressi*” OR “MDD”) AND (“prevalen*”) | 295 |
|  | (“ASD” OR “autis*” OR “Asperger”) AND (“depressi*” OR “MDD”) AND (“prevalen*”) | 182 |
| Shared genes and environment | (“ADHD” OR “attention deficit hyperactivity disorder” OR “hyperkinetic” OR “ASD” OR “autis*”) AND (“depressi*” OR “MDD”) AND (“genetic” OR “GWAS” OR “gene-environment correlation” OR “polygenic risk score”) | 1480 |
| Direct causal effects | ("ADHD" OR "attention deficit hyperactivity disorder" OR "hyperkinetic" OR "ASD" OR "autis*" OR “Asperger”) AND ("depressi*" OR "MDD") AND ("causal" OR "cause" OR "causes") | 242 |
| Third disorder | (“ADHD” OR “attention deficit hyperactivity disorder” OR “hyperkinetic” OR "ASD" OR "autis*" OR “Asperger”) AND (“depressi*” OR “MDD”) AND (“epiphenomenal” OR “comorbid*” OR “anxiety” OR “OCD” OR “social anxiety” OR “alexithymia” OR “ODD” OR “irritab*” OR “conduct”) | 2225 |
| Social and psychological mechanisms | ("ADHD" OR "attention deficit hyperactivity disorder" OR "hyperkinetic") AND ("depressi*" OR "MDD") AND ("social" OR "psych*" OR "mechanism*" OR "pathway*" OR "mediat*") | 1331 |
|  | ("ASD" OR "autis*" OR "Asperger") AND ("depressi*" OR "MDD") AND ("social" OR "psych*" OR "mechanism*" OR "pathway*" OR "mediat*") | 1261 |
| Presentation | ("ADHD" OR "attention deficit hyperactivity disorder" OR "hyperkinetic") AND ("depressi*" OR "MDD") AND ("onset” OR "traject*" OR "symptom*") | 961 |
|  | (“ASD” OR “autis*” OR “Asperger”) AND (“depressi*” OR “MDD”) AND (“onset” AND/OR “traject*” AND/OR “symptom*”) | 543 |
| Assessment | (“ADHD” OR “attention deficit hyperactivity disorder” OR “hyperkinetic”) AND (“depress*” OR “MDD”) AND (“assess*” OR “measure*” OR “informant” OR “rate*” OR “report*”) | 1337 |
|  | ("ASD" OR "autis*" OR "Asperger") AND ("depressi*" OR "MDD") AND (“assess*” OR “measure*” OR “informant” OR “rate*” OR “report*”) | 1051 |
| Psychological treatment | (“ADHD” OR “attention deficit hyperactivity disorder” OR “hyperkinetic” OR "ASD" OR "autis*" OR “Asperger”) AND (“depress*” OR “MDD”) AND (therap* OR psycholog* OR treatment) | 995 |
| Medication treatment | (“ADHD” OR “attention deficit hyperactivity disorder” OR “hyperkinetic” OR “ASD” OR “autis*”) AND (“depressi*” OR “MDD”) AND (“antidepressant” OR “medication”) | 1532 |

**References**

Althoff, R. R., Kuny-Slock, A. V., Verhulst, F. C., Hudziak, J. J., & Van Der Ende, J. (2014). Classes of oppositional-defiant behavior: concurrent and predictive validity. *Journal of Child Psychology and Psychiatry*, *55*(10), 1162–1171.

Biederman, J., Ball, S. W., Monuteaux, M. C., Mick, E., Spencer, T. J., McCreary, M., Cote, M., et al. (2008). New insights into the comorbidity between ADHD and major depression in adolescent and young adult females. *Journal of the American Academy of Child and Adolescent Psychiatry*, *47*(4), 426–434.

Bird, G., & Cook, R. (2013). Mixed emotions: The contribution of alexithymia to the emotional symptoms of autism. *Translational Psychiatry*, *3*(7), e285.

Blackman, G. L., Ostrander, R., & Herman, K. C. (2005). Children with ADHD and depression: a multisource, multimethod assessment of clinical, social, and academic functioning. *Journal of Attention Disorders*, *8*(4), 195–207.

Chen, T. J., Ji, C. Y., Wang, S. S., Lichtenstein, P., Larsson, H., & Chang, Z. (2016). Genetic and environmental influences on the relationship between ADHD symptoms and internalizing problems: A Chinese twin study. *American Journal of Medical Genetics, Part B: Neuropsychiatric Genetics*, *171*(7), 931–937.

Chronis-Tuscano, A., Molina, B. S. G., Pelham, W. E., Applegate, B., Dahlke, A., Overmyer, M., & Lahey, B. B. (2010). Very early predictors of adolescent depression and suicide attempts in children with attention-deficit/hyperactivity disorder. *Archives of General Psychiatry*, *67*(10), 1044–1051.

D’Agati, E., Curatolo, P., & Mazzone, L. (2019). Comorbidity between ADHD and anxiety disorders across the lifespan. *International Journal of Psychiatry in Clinical Practice*, *23*(4), 238–244.

Dalsgaard, S., Mortensen, P. B., Frydenberg, M., Maibing, C. M., Nordentoft, M., & Thomsen, P. H. (2014). Association between attention-deficit hyperactivity disorder in childhood and schizophrenia later in adulthood. *European Psychiatry*, *29*(4), 259–263.

Davidsson, M., Hult, N., Gillberg, C., Särneö, C., Gillberg, C., & Billstedt, E. (2017). Anxiety and depression in adolescents with ADHD and autism spectrum disorders; correlation between parent- and self-reports and with attention and adaptive functioning. *Nordic Journal of Psychiatry*, *71*(8), 614–620.

Daviss, W.B., Birmaher, B., Melhem, N. A., Axelson, D. A., Michaels, S. M., & Brent, D. A. (2006). Criterion validity of the Mood and Feelings Questionnaire for depressive episodes in clinic and non‐clinic subjects. *Journal of Child Psychology and Psychiatry*, *47*(9), 927–934.

Ghaziuddin, M., & Ghaziuddin, N. (2021). Bipolar disorder and psychosis in autism. *Child and Adolescent Psychiatric Clinics of North America*, *29*(3), 433–441.

Hollocks, M. J., Lerh, J. W., Magiati, I., Meiser-Stedman, R., & Brugha, T. S. (2019). Anxiety and depression in adults with autism spectrum disorder: A systematic review and meta-analysis. *Psychological Medicine*, *49(*4), 559–572.

Hu, H.-F., Liu, T.-L., Hsiao, R. C., Ni, H.-C., Liang, S. H.-Y., Lin, C.-F., Chan, H.-L., et al. (2019). Cyberbullying victimization and perpetration in adolescents with high-functioning Autism Spectrum Disorder: Correlations with depression, anxiety, and suicidality. *Journal of Autism and Developmental Disorders*, *49*(10), 4170-4180.

Kowatch, R. A. (2016). Diagnosis, phenomenology, differential diagnosis, and comorbidity of pediatric bipolar disorder. *The Journal of Clinical Psychiatry*, *77*(Suppl E1), e01.

López-Pinar, C., Martínez-Sanchís, S., Carbonell-Vayá, E., Sánchez-Meca, J., & Fenollar-Cortés, J. (2020). Efficacy of nonpharmacological treatments on comorbid internalizing symptoms of adults With Attention-Deficit/Hyperactivity Disorder: A meta-analytic review. *Journal of Attention Disorders*, *24*(3), 456–478.

Margari, L., Palumbi, R., Peschechera, A., Craig, F., De Giambattista, C., Ventura, P., & Margari, F. (2019). Sex-gender comparisons in comorbidities of children and adolescents with high-functioning Autism Spectrum Disorder. *Frontiers in Psychiatry*, *10*.

Martin, F., & Oliver, T. (2019). Behavioral activation for children and adolescents: a systematic review of progress and promise. *European Child & Adolescent Psychiatry*, *28*(4), 427–441.

McCauley, J. B., Elias, R., & Lord, C. (2020). Trajectories of co-occurring psychopathology symptoms in autism from late childhood to adulthood. *Development and Psychopathology*, *32*(4), 1287–1302.

Melton, T. H., Croarkin, P. E., Strawn, J. R., & Mcclintock, S. M. (2016). Comorbid anxiety and depressive symptoms in children and adolescents: A Systematic review and analysis. *Journal of Psychiatric Practice*, *22*(2), 84–98.

Montes, G. (2018). Having older siblings is associated with lower rates of depression, ADD/ADHD, anxiety and behavior problems among children with ASD. *Maternal and Child Health Journal*, *22*(5), 642–647.

Nasca, B. C., Lopata, C., Donnelly, J. P., Rodgers, J. D., & Thomeer, M. L. (2020). Sex differences in externalizing and internalizing symptoms of children with ASD. *Journal of Autism and Developmental Disorders*, *50*(9), 3245–3252.

Nøvik, T. S., Hervas, A., Ralston, S. J., Dalsgaard, S., Rodrigues Pereira, R., Lorenzo, M. J., Baldursson, G., et al. (2006). Influence of gender on attention-deficit/hyperactivity disorder in Europe--ADORE. *European Child & Adolescent Psychiatry*, *15,* i1–i24.

Poquérusse, J., Pastore, L., Dellantonio, S., & Esposito, G. (2018). Alexithymia and Autism Spectrum Disorder: A complex relationship. *Frontiers in Psychology*, *9*, 1196.

Rucklidge, J. J., & Tannock, R. (2001). Psychiatric, psychosocial, and cognitive functioning of female adolescents with ADHD. *Journal of the American Academy of Child and Adolescent Psychiatry*, *40*(5), 530–540.

Russell, A., Gaunt, D. M., Cooper, K., Barton, S., Horwood, J., Kessler, D., Metcalfe, C., et al. (2020). The feasibility of low-intensity psychological therapy for depression co-occurring with autism in adults: The Autism Depression Trial (ADEPT)–a pilot randomised controlled trial. *Autism*, *24*(6), 1360–1372.

Schiltz, H. K., McVey, A. J., Dolan Wozniak, B., Haendel, A. D., Stanley, R., Arias, A., Gordon, N., et al. (2021). The role of loneliness as a mediator between autism features and mental health among autistic young adults. *Autism*, *25*(2), 545–555.

Shaw, P., De Rossi, P., Watson, B., Wharton, A., Greenstein, D., Raznahan, A., Sharp, W., et al. (2014). Mapping the development of the basal ganglia in children with attention-deficit/hyperactivity disorder. *Journal of the American Academy of Child and Adolescent Psychiatry*, *53*(7), 780–789.

Skogli, E. W., Teicher, M. H., Andersen, P. N., Hovik, K. T., & Øie, M. (2013). ADHD in girls and boys--gender differences in co-existing symptoms and executive function measures. *BMC Psychiatry, 13*, 298.

Spek, A. A., van Ham, N. C., & Nyklíček, I. (2013). Mindfulness-based therapy in adults with an autism spectrum disorder: A randomized controlled trial. *Research in Developmental Disabilities*, *34*(1), 246–253.

Stewart, M. E., Barnard, L., Pearson, J., Hasan, R., & O’Brien, G. (2006). Presentation of depression in autism and Asperger syndrome. *Autism*, *10*(1), 103–116.

Sullivan, P. F., Neale, M. C., & Kendler, K. S. (2000). Genetic epidemiology of major depression: Review and meta-analysis. *The American Journal of Psychiatry*, *157*(10), 1552–1562.

Tandon, M., Cardeli, E., & Luby, J. (2009). Internalizing disorders in early childhood: A review of depressive and anxiety disorders. *Child and Adolescent Psychiatric Clinics of North America*, *18*(3), 593–610.

Thapar, A., Cooper, M., Eyre, O., & Langley, K. (2013). Practitioner review: What have we learnt about the causes of ADHD? *Journal of Child Psychology and Psychiatry*, *51*(1), 3–16.

Thapar, A., & Rutter, M. (2015). Using natural experiments and animal models to study causal hypotheses in relation to child mental health problems. In A. Thapar, D. S. Pine, J. F. Leckman, S. Scott, M. J. Snowling, & E. Taylor (Eds.), *Rutter’s child and adolescent psychiatry, 6^th^ edn* (pp. 143–162). Chichester, UK: John Wiley and Sons Ltd.

Tsujii, N., Usami, M., Naya, N., Tsuji, T., Mishima, H., Horie, J., Fujiwara, M., et al. (2021). Efficacy and safety of medication for Attention-Deficit Hyperactivity Disorder in children and adolescents with common comorbidities: A systematic review. *Neurology and Therapy*, *10*(2), 499–522.

Uljarević, M., Hedley, D., Rose-Foley, K., Magiati, I., Cai, R. Y., Dissanayake, C., Richdale, A., et al. (2020). Anxiety and depression from adolescence to old age in Autism Spectrum Disorder. *Journal of Autism and Developmental Disorders*, *50*(9), 3155–3165.

Vasa, R. A., Keefer, A., Reaven, J., South, M., & White, S. W. (2018). Priorities for advancing research on youth with Autism Spectrum Disorder and co-occurring anxiety. *Journal of Autism and Developmental Disorders*, *48*(3), 925–934.

Warrier, V., Kwong, A. S. F., Luo, M., Dalvie, S., Croft, J., Sallis, H. M., Baldwin, J., et al. (2021). Gene-environment correlations and causal effects of childhood maltreatment on physical and mental health: a genetically informed approach. *The Lancet Psychiatry*, *8*(5), 373–386.

Weersing, V. R., Shamseddeen, W., Garber, J., Hollon, S. D., Clarke, G. N., Beardslee, W. R., Gladstone, T. R., et al. (2016). Prevention of depression in at-risk adolescents: Predictors and moderators of acute effects. *Journal of the American Academy of Child and Adolescent Psychiatry*, *55*(3), 219–226.

Yücwe, M., Zoroglu, S. S., Ceylan, M. F., Kandemir, H., & Karabekiroglu, K. (2013). Psychiatric comorbidity distribution and diversities in children and adolescents with attention deficit/hyperactivity disorder: A study from Turkey. *Neuropsychiatric Disease and Treatment*, *9*, 1791–1799.
